# Edible bird’s nest modulate intracellular molecular pathways of influenza A virus infected cells

**DOI:** 10.1186/s12906-016-1498-x

**Published:** 2017-01-05

**Authors:** Amin Haghani, Parvaneh Mehrbod, Nikoo Safi, Fadzilah A’ini Abd Kadir, Abdul Rahman Omar, Aini Ideris

**Affiliations:** 1 Institute of Bioscience, Universiti Putra Malaysia, 43400 Serdang, Selangor Malaysia; 2 Faculty of Veterinary Medicine, Universiti Putra Malaysia, 43400 Serdang, Selangor Malaysia; 3 Davis School of Gerontology, University of Southern California, Los Angeles, CA USA

**Keywords:** Edible bird nest (EBN), Influenza A virus, Antiviral, Sialic acid, Immunoflourescent, Autophagy, Endosome trafficking

## Abstract

**Background:**

Edible Bird’s Nest (EBN) as a popular traditional Chinese medicine is believed to have health enhancing and antiviral activities against influenza A virus (IAV); however, the molecular mechanism behind therapeutic effects of EBN is not well characterized.

**Methods:**

In this study, EBNs that underwent different enzymatic preparation were tested against IAV infected cells. 50% cytotoxic concentration (CC_50_) and 50% inhibitory concentration (IC_50_) of the EBNs against IAV strain A/Puerto Rico/8/1934(H1N1) were determined by HA and MTT assays. Subsequently, the sialic acid content of the used EBNs were analyzed by fluorometric HPLC. Western Blotting and immunofluorescent staining were used to investigate the effects of EBNs on early endosomal trafficking and autophagy process of influenza virus.

**Results:**

This study showed that post inoculations of EBNs after enzymatic preparations have the highest efficacy to inhibit IAV. While CC50 of the tested EBNs ranged from 27.5–32 mg/ml, the IC50 of these compounds ranged between 2.5–4.9 mg/ml. EBNs could inhibit IAV as efficient as commercial antiviral agents, such as amantadine and oseltamivir with different mechanisms of action against IAV. The antiviral activity of these EBNs correlated with the content of *N*-acetyl neuraminic acid. EBNs could affect early endosomal trafficking of the virus by reducing Rab5 and RhoA GTPase proteins and also reoriented actin cytoskeleton of IAV infected cells. In addition, for the first time this study showed that EBNs can inhibit intracellular autophagy process of IAV life cycle as evidenced by reduction of LC3-II and increasing of lysosomal degradation.

**Conclusions:**

The results procured in this study support the potential of EBNs as supplementary medication or alternative to antiviral agents to inhibit influenza infections. Evidently, EBNs can be a promising antiviral agent; however, these natural compounds should be screened for their metabolites prior to usage as therapeutic approach.

## Background

For centuries, influenza epidemics and pandemics have caused enormous mortality and economical costs for human and various animal species [1, 2]. As opposed to the number of attempts to control influenza, currently, there is no effective control measure to counteract this devastating disease. Since vaccines only can be available several months after occurrence of influenza pandemics, antiviral agents can be considered as the first line of defense against this virus. However, widespread resistance of influenza A virus (IAV) strains against the current antiviral agents has emphasized the necessity of development of new preventive measures [3]. Consequently, scientists started to study several molecular targets that can be used against influenza viruses, and in a mean while ameliorate pathogenesis and prognosis of this disease [3, 4]. Some of these novel targets can be illustrated by molecular pathways related to influenza virus entry (e.g. Epsin-1, RTK cascades, Rab5, endosomal autoantigen 1 (EEA1) and PI3K), and some potential targets during intracellular autophagy process of influenza viruses (e.g. mTOR signaling pathways, LC3 and class III phosphatidylinositol 3-kinase (PI3K) complex) [5, 6]. IAV entry to the host cell is a dynamic action with several steps that includes attachment of viral hemagglutinin (HA) with sialic acid receptors of the host cell, internalization by receptor-mediated endocytosis (or macropinocytosis), trafficking of endosomes to perinuclear regions, fusion with endosomal membranes, release of viral ribonucleoprotein complex (vRNP) into the cytoplasm, and transport of these vRNPinto the host nucleus [5]. After entry and during the replication, influenza virus would start to exploit the host autophagy pathway by controlling of the fusion of lysosomes to the autophagosomes, which would accelerate the virus replication, reduce antigenicity of the infected cells to evade the host immune system and manipulate apoptosis process of the host cells depend on the state of the infection [6, 7]. Therefore, all of these molecular pathways can be possible targets for inhibiting IAV replication and controlling of the disease.

Previous studies have shown that edible bird’s nest (EBN), a popular Chinese traditional medicine, can inhibit the hemagglutination activity of influenza viruses [8–10]. These nests are mainly produced by two species, which include white nest swiftlet (*Aerodramus fuciphogus*) and black nest swiftlet (*Aerodramus maximus*) [11]. This natural medicine composed of more than 78 metabolites from carbohydrates, proteins, fatty acids, hormones and minerals [12]. This remedy is used for several purposes like dissolving phlegm, voice improvement, libido raising, gastric problems, renal dysfunctions, asthma, cough and tuberculosis to name but a few in Chinese medicine and cuisine [13]. In past decades, scientists have shown many properties of EBNs such as cell proliferation induction by epidermal growth factor (EGF) like components, immunomodulatory effects such as decrease of TNFα and nitric oxide (NO) generation in RAW cells, improvement of corneal keratocyte proliferation which can be helpful in wound healing, neuroprotective activity against 6-hydroxydopamine (6-OHDA)-induced degeneration of dopaminergic neurons, improvement of bone strength and skin thickness, chondro protective properties by reduction of catabolic activities and potential antiviral activity against influenza viruses [8, 10, 14–18]. Despite of these studies, potential bioactivities of EBN’s components require subsequent researches and characterization of the mechanisms of action that can provide valuable information on their usage in orthodox medicine.

Consequently, the current study was designed to evaluate the antiviral properties of EBN against two of the aforementioned molecular pathways in IAV life cycle. At first, the cytotoxic and antiviral activity of EBNs with different enzymatic treatments against IAV was determined. Later, this study focused on the effects of EBN on early endosomal trafficking of IAV by evaluating actin cytoskeleton and small GTPAse proteins such as Rab5 as early endosomes biomarker and RhoA that involve in actin filaments polymerization. Finally, intracellular autophagy process was assessed by LC3-II protein as a marker for autophagosomes accumulation and immunoflourescent staining of lysosomes for lysosome activity.

## Methods

### Reagents and chemicals

The materials of this study were purchased from different sources as follows: Dulbecco’s modified Eagle’s medium (DMEM) and penicillin-streptomycin solution from Mediatech Cellgro Company (Northbrook, Illinois, USA), Accutase® cell detachment solution from Innovative Cell Technologies (San Diego, California, USA), fetal bovine serum (FBS) from PAA Laboratories (Pasching, Austria). Plastic wares (Orange Scientific, Braine-l’Alleud, Belgium), Lab-Tek II Chamber Slides (8-well) (Thermo scientific, New York, USA) were used during the experiments. Amantadine hydrochloride, oseltamivir carboxylate, Tosylamide phenylethyl chloromethyl keton-treated trypsin (TPCK-Trypsin), 3-(4, 5-dimethyl-2-thiazolyl)-2, 5- diphenyl-2H-tetrazolium bromide (MTT), fluorescent rhodamine 110 phalloidin, RhoA inhibitor,Y-27632 dihydrochloride and lysosome inhibitor,Bafilomycin A1 (BafA1) were procured from Sigma (Saint Louis, Missouri, USA). Rab5 inhibitor, NE10790was provided by Dr. David F. Wiemer, Department of Chemistry, University of Iowa, USA. Pancreatin F and neuraminidase were purchased from Sigma, USA. ProLong® Gold Antifade reagent and LysoTracker Red DND-99 were obtained from Invitrogen (Carlsbad, California, USA).

Protein extraction kit (ab65400), rabbit polyclonal anti-Rab5 antibody (ab18211), rabbit polyclonal anti-RhoA antibody (ab68826), rabbit polyclonal anti-LC3A/B antibody (ab58610), mouse monoclonal anti-pan cadherin antibody [CH-19] (ab6528) and goat polyclonal anti-mouse IgG H&L (alkaline phosphate conjugated) (ab97020) were procured from Abcam (Cambridge, USA).

### Virus and cell culture

Influenza virus strain used in this study, A/Puerto Rico/8/1934 (H1N1) (ATCC VR-1469™), and Madin Darby Canine Kidney (MDCK) cell line (CCL-34™) were acquired from ATCC. The viral stocks were propagated in MDCK cells in the presence of 0.5 μg/ml of TPCK-Trypsin. DMEM containing 10% heat-inactivated FBS, 100 units/ml penicillin G and 100 μg/ml streptomycin was used as growing media. Incubation conditions were 37 °C with 5% CO_2_. DMEM supplemented with TPCK-Trypsin (0.5 μg/ml) was used as maintenance media during antiviral experiments. During virus-stock preparation, virus infected MDCK cells at three days post-infection were used. Tissue culture infectious dose 50 (TCID_50_) in combination with hemagglutination assay were used to measure virus infectivity dose in different treatments [19, 20].

### Preparation of EBN extracts

EBN extracts were prepared based on enzymatic and heat treatments as described in a previous study with some modification [8]. House nest EBN from Teluk Intan, Perak, Malaysia and cave nest EBN from Gua Madai caves, Lahad Datu, Sabah, Malaysia were collected by our research team (Dr. Kadir and Dr. Mehrbod) and formally identified by Dr. Fadzilah A’ini, Kadir.A (Chairman of ranching Edible-Nest Swiftlet, Malaysia Standards (MS)). The collected EBNs were prepared in four types as follows: 1) House nest EBN from Teluk Intan of Perak, Malaysia, with no enzymatic treatment, 2) House nest EBN from TelukIntan of Perak, Malaysia, with pancreatin F-treatment, 3) Cave nest EBN from Gua Madai of Sabah, Malaysia, with no enzymatic treatment, 4) Cave nest EBN from Gua Madai of Sabah, Malaysia, with neuraminidase-treatment. For preparation, first, nests were dried at 70 °C for 16 h. Afterwards, they were grounded and filtered with 600 μm in pore size mesh to remove the feathers and foreign substances. Later, 5 g of each sample were suspended in 200 ml dH2O for 16 h at 5°Cfollowed by heating for around 1 h at 60 °C. Afterwards some of the samples were treated with 0.5 mg/ml of pancreatin F for 4 h at 45 °C with pH 8.5–9.0. Subsequently, suspensions were heated at 90 °C for 5 min for enzyme deactivation. The treated extracts were filtered using filter papers (ADVANTEC filter paper no. 2) and after 48 h freezing at −80 °C the filtrate was freeze-dried, and then stored at −80 °C for further use. Samples that were treated with neuraminidase (from *Clostridium perfringens*) (N2876, Sigma) also followed the same process except for the incubation at 37 °C for 2 h. A working concentration of 0.05 g/ml of dH2O was used for cell culture exposure.

### Hemagglutination-inhibitione test

Briefly, EBNs were two-fold serially diluted in round-bottomed wells in 96-well microtitre plates (Nunc, Denmark). From the virus stock, 4HA unit (the lowest amount of virus particles able to agglutinate the chicken erythrocytes) was added to all wells (25 μl/well) to investigate the inhibitory effect of EBNs onto the hemagglutinating activity. After pre-incubation of 45 min at room temperature (R.T.), chicken erythrocytes (taken from chicken, washed with PBS at least 3 times and diluted to 1%) were mixed with the solution. Following 1 h incubation at R.T. the agglutination inhibition pattern was read.

### Cytotoxicity evaluation of EBNs

To determine possible cytotoxic effects of EBN extracts, monolayer MDCK cells in 96-well plates were exposed to different concentrations of EBN for 48 h with at least six replications and cell viability was measured by methylthiazolyl- diphenyl-tetrazolium bromide (MTT) viability assay as described by Mehrbod et al. [20]. Briefly, cell monolayers were washed after 48 h exposure period and 100 μl MTT 1X in Phosphate buffered saline (PBS) was added to each well and the plates were further incubated at 37 °C in CO_2_ incubator for 2–3 h. Solubilization of the formed formazan crystals during this period was achieved by the addition of DMSO solution. Color adsorption (OD) was evaluated by microplate reader (BioTek EL 800, US) at 570 nm. Cell survival rate was determined as average OD of treatment value/average OD of control value. Accordingly, the 50% cytotoxic concentration (CC_50_) causing cytopathic effects in half of the cells as a ratio to control cell and maximum non-cytotoxic concentration (MNCC) of EBNs were determined.

### EBN extracts antiviral activity

For antiviral assessment of EBNs, semi-confluent monolayer MDCK cells in 96-well plates were exposed to EBN extracts (MNCC) and IAV (100 TCID_50_/0.1 ml) in three different exposure types. Briefly, MDCK cells were treated with EBNs for 1 h, which were followed by 1 h incubation with virus for infection (pre-treatment assay), adding EBNs and viruses together to the monolayer cells with 1 h incubation period (co-treatment assay), or treatment of already infected cells with EBNs for 1 h period (post-treatment assay). Afterwards, cells were covered with medium containing TPCK-Trypsin (0.5 μg/ml) for 48 h. Subsequently, the viability of the cells was defined by MTT assay and virus titers were determined by hemagglutination assay [21]. During all antiviral activity tests, cell viability and viral load of non-treated cells and non-infected cells treated with EBNs or antiviral agents were considered as the negative controls for comparison and reliability confirmation of each experiment. Later, percentage of protection of each treatment was calculated by the following formula:

$$ \mathrm{Percentage}\kern0.5em \mathrm{of}\kern0.5em \mathrm{protection}=\frac{A-B}{C-B} $$

A: Absorbance of the samples

B: Absorbance of the virus-infected control

C: Absorbance of negative control of both virus and compound

Since the results showed that post-treatment of IAV with EBNs had the highest protection, this treatment approach is further analyzed by incubating the infected cells with EBNs for 1, 8 & 24 h. The cell viability and virus titers were determined at 48 h post infection time point by MTT and HA assays. In the last step, 50% inhibitory concentration (IC_50_) of EBNs were determined by treating the infected cells with serial dilution of each EBN, followed by HA assay for determination of virus inhibition in each treatment. Later, the selectivity index (therapeutic index) of each EBN was calculated as a ratio of CC_50_/IC_50_ for each compound.

### Fluorometric HPLC analysis of EBNs for sialic acid contents

In this step, total 5-*N*-acetylneuraminic acid (Neu5Ac) contents of the prepared EBNs from Gua Madai and Teluk Intan with no enzymatic treatments were analyzed by fluorometric high performance liquid chromatography (HPLC) as described previously [22]. Briefly, 5 mg of samples were solved in sodium hydrogen sulphate solution and incubated at 80 °C for 30 min. Afterwards, the sialic acids were derivetized by o-Phenylenediamine dihydrochloride (Sigma, USA) and separated on a C_18_ column by a mobile phase composed of 1.0% tetrahydrofuran aqueous and acetonitrile at 1.0 ml/min flow rate in an isocratic elution. The fluorescent intensities were analyzed at 230 nm excitation and 425 emission wavelengths. Finally, sialic acids were identified and quantified using the elution position and standard curve of Neu5Ac standard (Sigma, USA).

### Sample preparation for immunoblotting

For this step, MDCK cells were cultured in the required number in 75 cm^2^ flasks (Orange Scientific, Belgium) for different treatments. Then, the infected (100 TCID_50_/0.1 ml) and non-infected semi confluent flasks were treated with EBNs or anti-influenza drugs as described above. After 48 h incubation, the cells were scraped, collected in chilled PBS and centrifuged to form pellet. Then, the cell pellets were suspended in 1 ml of cytobuster™ protein extraction reagent (Novagen, USA) containing 2% protease inhibitor cocktail from Protein extraction kit (ab65400) (Abcam, UK). After vortexing with beads for few minutes, the lysates were centrifuged for 3 min at 825 × g. 4 °C. The supernatants were transferred to new microtubes and stored at −80 °C till further usage. The concentration of the proteins of the samples were assessed by Bradford assay [23] and all the concentrations were normalized to 1 μg/μl by dilution with PBS.

### Detection of Rab5, RhoA and LC3 translocation in MDCK cells

For Western blotting, 1 μg of proteins of each sample was fractionized by sodium dodecyl sulfate polyacrylamide gel electrophoresis (SDS-PAGE) and electrophoretic transfer of the bonds to nitrocellulose membrane (Bio-Rad, USA) using vertical semi-dry electroblotting. Subsequently, the membranes were blocked by milk diluent as blocking buffer (Kierkegaard and Perry Laboratories, Gaithersburg, USA) for 1 h at room temperature. Then, the membranes were washed for three times with Tris Buffered Saline (TBS)-tween buffer, which followed by incubation with specific primary antibody at 4 °C overnight. In the next day, the membranes were washed for few times and incubated with alkaline phosphatase-conjugated antibody at room temperature for 1 h. This was followed by three times washing and visualizing of the bands by enzymatic reaction with BCIP-NBT substrate (Sigma, USA). Subsequently, the bands were scanned with Odyssey infrared imaging system (Li-COR Bioscience, Nebraska, USA) at 21–339 μm scanning range. The intensity of the bands was quantified on a single channel. Pan-Cadherin protein was used as house-keeping protein to normalize the result of each sample.

### Immunofluorescent labelling of actin cytoskeleton and lysosomal bodies

MDCK cells cultured up to 80% confluent in 8-well chamber-slides (8 × 10^4^ cell/well) for 24 h. Then, influenza infected (100 TCID_50_/0.1 ml) or non-infected cells were treated with EBN extracts as described above. For visualization of actin cytoskeleton, the cells were fixed by fresh 3% paraformaldehyde in PBS for 10 min at 4 °C at the end of the incubation period of 24 h. This was followed by permeabilization with 0.2% Triton-X-100 at room temperature, washing with PBS and then blocking with 10% FBS in PBS for 1 h at 4 °C. Finally, rhodamine 110 phalloidin stain with 2 μM concentration (1:50 dilution in blocking buffer) were used for 20 min at room temperature to label the cells. For detection of lysosome, the live treated cells were incubated with pre-warmed media containing 50 nM LysoTracker Red DND-99 for 25 min at 24 h post infection time point, which was followed by fixation with 3% paraformaldehyde as before. At the end, all the labeled slides was mounted with warm anti-fade reagent containing 4′,6-diamidino-2-phenylindole (DAPI) (1 μg/ml) as nuclear counter-stain and sealed with nail polish. Fluorescent images were acquired using confocal microscopy (Olympus, Japan) using suitable filters (502 nm excitation and 524 nm emission for rhodamine 110 phalloidin, 577 nm excitation and 592 nm emission for LysoTracker, and 358 nm excitation and 461 nm emission for DAPI) on 100X lens, 0.164 μm pixel resolution, and scanning at speed of 5 with averaging of four lines from an argon–krypton laser. The images were processed using Flouview ver3.1a and ImageJ64 software.

### Statistical analysis

Data statistical analyses, which were expressed as mean ± SD, were performed using Statistical Package for the Social Sciences (SPSS 22.0). One-way analysis of variance (ANOVA) post-hoc Dunkan test were used to evaluate the significance of differences (*p* < 0.05) among treatments. For the data with two continuous variables, like CC_50_ and IC_50_ determination, linear regression was used to calculate these values by respective prediction formulas. Lastly, the graphs with the respective analysis were drawn by Graphpad Prism version 5.0 software.

## Results

### Cytotoxicity results

As shown in Table 1, EBNs from Teluk Intan showed lower CC_50_ (27.5–30.5 mg/ml) in contrast to the ones from Gua Madai (31.5–32 mg/ml). Both enzymatic treatments increased the CC_50_ concentration of the treated EBN; however, pancreatin F decreased the toxic effects of EBN more efficiently. The highest concentration with no significant cytotoxicity, which was considered as MNCC of EBNs for following experiments, was 12.5 mg/ml in all EBNs (Table 1). CC_50_ of amantadine in MDCK cells was 4523.4 mg/ml, which is equal to 24.09 mol/L. This value was 14373.2 mg/ml (35.02 mol/L) for oseltamivir in MDCK cells. The results showed that 1510.0 mg/ml (8.04 mol/L) of amantadine and 3120.0 mg/ml (7.60 mol/L) of oseltamivir is the highest concentrations with no cytotoxicity for MDCK cells, which were used as MNCC of these drugs in the following steps.

**Table 1:** CC_50_, IC_50_ and selectivity index of different EBN sources and enzymatic treatments

	EBN1	EBN2	EBN3	EBN4
CC_50_ (mg/ml)	27.5	30.5	31.5	32
IC_50_ (mg/ml)	4.9	3.6	2.5	3.6
Selectivity index	5.61	8.47	12.60	8.89

*EBN1* EBN from Teluk Intan with no enzymatic treatment, *EBN2* EBN from Teluk Intan with Pancreatin F treatment, *EBN3* EBN from Gua Madai with no enzymatic treatment, *EBN4* EBN from Gua Madai with neuraminidase treatment

### Hemagglutination-inhibition results

Untreated erythrocytes were precipitated to the bottom of the wells, while upon pre-incubation with virus the blood cells showed even and diffuse distribution. EBN dilutions did not inhibit RBC agglutination at all, arguing against physical interaction of the EBN ingredients with RBC. Treatment of influenza virus with EBN reduced the heamagglutination activity of the virus, which indicates physical interaction of EBNs with virus hemagglutinin up to a certain dilution (dose-dependent manner).

### EBN extracts inhibitory effect on influenza A virus

The results of both HA and MTT assays for antiviral activity of EBNs against IAV showed that post treatment of influenza with EBN significantly (*p* < 0.05) reduced the virus titer and increased the cell viability (Table 2). While all the EBN samples showed significant antiviral activity, the EBN from Gua Madai with no enzymatic treatment (EBN3) showed the highest antiviral activity by reducing the virus HA titer from 1:128 to 1:10–1:20. In addition, the results showed that pancreatin F could not increase the antiviral activity of EBN. However, neuraminidase treatment of EBNs reduced the antiviral activity by increasing the virus HA titer from 1:10 to 1:20 and decreasing the percentage of protection of these compounds from 61 to 54% (Table 2). As opposed to the effects of EBNs, co-inoculation of amantadine and oseltamivir showed the highest efficiency by reduction of 1:128 virus HA titer to 1:5–1:10 (Table 2). Regarding the percentage of protection, EBN3 had the highest protection compared to the other EBNs. The results revealed that post-inoculation of EBN from Gua Madai with no enzymatic treatment (EBN3) could protect more than 60% of the infected cells compared to other EBNs (42 to 53% of protection), amantadine (39% of protection) and oseltamivir (40% of protection) (Table 2).

**Table 2:** Antiviral effects of EBNs and anti-influenza drugs on IAV infected MDCK cells

	Groups	OD for cell viability (Mean ± SD)	Virus titer Log_2_ (Mean ± SD)	Percentage of protection (%) (Mean ± SD)
Co inoculation	E1 + PR	0.58 ± 0.17	6 ± 0	31.52 ± 24.81^abcd^
	E2 + PR	0.55 ± 0.1	6.67 ± 0.58	27.98 ± 14.62^abc^
	E3 + PR	0.59 ± 0.13	6.33 ± 0.58	33 ± 18.79^abcd^
	E4 + PR	0.53 ± 0.07	6.33 ± 0.58	22.65 ± 9.11^abc^
	AMA + PR	0.64 ± 0.01	3.33 ± 0.58*	39.9 ± 1.71^abcde^
	OSE + PR	0.63 ± 0.05	2.33 ± 0.58*	39.41 ± 8.14^abcde^
Pre inoculation	E1 + PR	0.53 ± 0.07	6.33 ± 0.58	24.72 ± 10.06^abc^
	E2 + PR	0.54 ± 0.06	6.67 ± 0.58	26.2 ± 8.97^abc^
	E3 + PR	0.61 ± 0.05	6.33 ± 0.58	35.66 ± 7.99^abcd^
	E4 + PR	0.65 ± 0.04*	6.33 ± 0.58	42.17 ± 5.67^bcde^
	AMA + PR	0.5 ± 0.06	6.33 ± 0.58	20.19 ± 8.41^ab^
	OSE + PR	0.52 ± 0.03	5.33 ± 0.58*	22.16 ± 5.19^ab^
Post inoculation	E1 + PR	0.66 ± 0.05*	5 ± 0*	42.47 ± 8^bcde^
	E2 + PR	0.67 ± 0.06*	5 ± 0*	45.42 ± 8.4^cde^
	E3 + PR	0.78 ± 0.19*	3.33 ± 0.58*	61.1 ± 27.4^e^
	E4 + PR	0.73 ± 0.10*	4.33 ± 0.58*	53.7 ± 15.54^de^
	AMA + PR	0.5 ± 0.07	6.33 ± 0.58	20.19 ± 10.9^ab^
	OSE + PR	0.52 ± 0.03	6.33 ± 0.58	18.71 ± 6.67^a^
Controls	PR (Control)	0.37 ± 0.19	7 ± 0	
	Negative control	1.04 ± 0.09*		
	EBN1 with no virus	0.8 ± 0.14*		
	EBN2 with no virus	0.81 ± 0.03*		
	EBN3 with no virus	0.87 ± 0.10*		
	EBN4 with no virus	0.8 ± 0.14*		
	Ama with no virus	0.86 ± 0.04*		
	Ose with no virus	0.89 ± 0.00*		

Note: The results are average of at least 6 replicates for each group

*E1* EBN from Teluk Intan with no enzymatic treatment, *E2* EBN from Teluk Intan with Pancreatin F treatment, *E3* EBN from Gua Madai with no enzymatic treatment, *E4* EBN from Gua Madai with neuraminidase treatment, *Ama* Amantadine hydrochloride, *Ose* Oseltamivir carboxylate, *PR* influenza A virus, A/Puerto Rico/8/1934(H1N1)

*Shows significant difference with infected control (PR) (*p* < 0.05). Different letters on the top of the percentage of protection data show the statistical grouping by Duncan analysis of the treatments (*p* < 0.05)

**Fig. 1:**
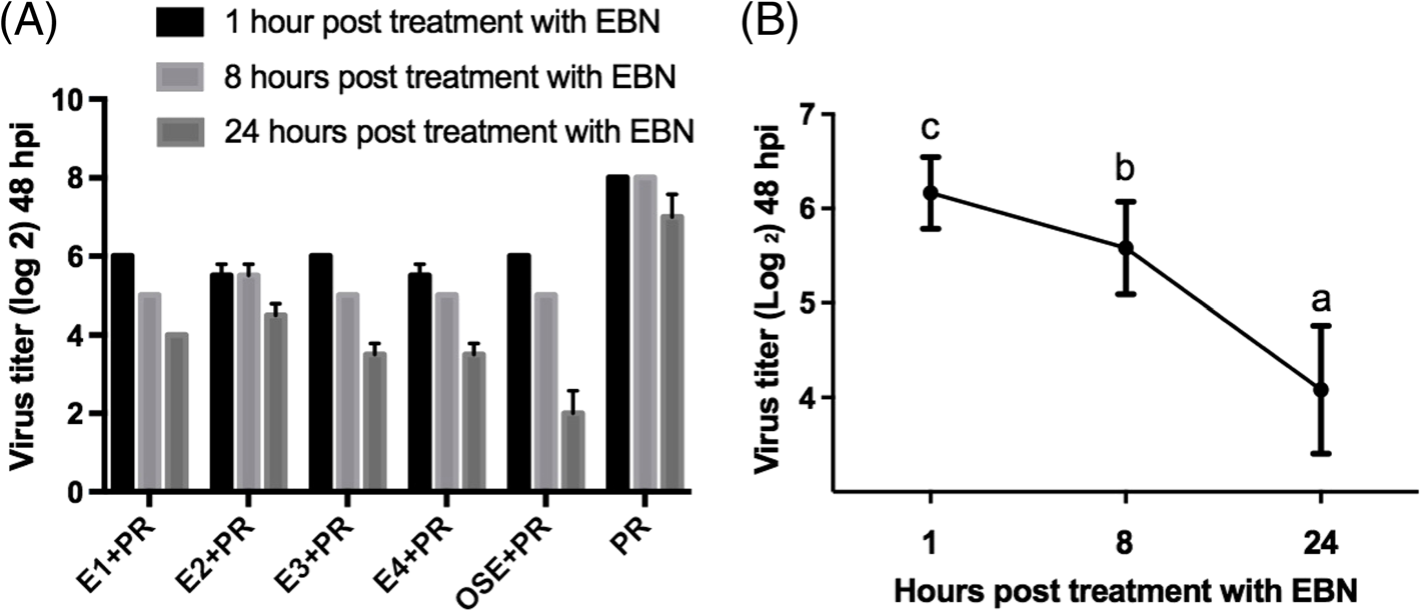
Effects of post-inoculation of EBNs at different incubation periods against IAV. **a** Statistical analysis of HA assay results at 48 hpi of the trend of antiviral activity of EBNs with different inoculation periods. Ose was tested as antiviral control drug. **b** The trend of change in virus titer at 48 hpi following different post infection inoculation of EBN. Alphabets show the significant (*p* < 0.05) differences among different treatments. E1: EBN from Teluk Intan with no enzymatic treatment, E2: EBN from Teluk Intan with Pancreatin F treatment, E3: EBN from Gua Madai with no enzymatic treatment, E4: EBN from Gua Madai with neuraminidase treatment, Ose: Oseltamivir carboxylate, PR: influenza A virus (A/Puerto Rico/8/1934 (H1N1)). The statistical method ANOVA and post hoc Duncan was used to analyze the significant difference (*p* < 0.05)

This study also showed that increasing the post treatment period of EBNs would significantly (*p* < 0.05) augment the antiviral activity of these compounds (Fig. 1). At 48 h post infection, the HA results showed that 24 h treatment of IAV (1:256 HA titer) with EBN can significantly (*p* < 0.05) reduce the HA titer from 1:32 to 1:11 in comparison with 1 h or 8 h EBN treatment durations. In contrast to HA titer, the viability of the cells did not significant changed with different treatment durations. In addition to EBNs, the results showed that 24 h treatment of infected cells with oseltamivir also significantly (*p* < 0.05) improved the antiviral activity of this compound. Since EBNs showed higher efficacy in this time point, 24 h treatment duration was chosen for the following steps of this study.

### IC_50_ and selectivity index results

Linear regression analysis of the HA results for IC_50_ determination showed that the IC_50_ of the EBNs ranged from 2.5–4.9 mg/ml. In the next step, the therapeutic (selectivity) index of the EBNs was calculated from the ratio of CC_50_ per IC_50_. The results showed that the EBN extract from Gua Madai with no enzymatic treatment (EBN3) has the highest selectivity index (12.6) compared to other EBNs. In general, EBNs from Gua Madai had lower IC_50_ (2.5–3.6 mg/ml) and higher selectivity index (8.89–12.6) than EBNs from Teluk Intan (Table 1). Meanwhile, pancreatin F treatment of EBN increased the selectivity index from 5.6 to 8.5, while neuraminidase treatment decreased this value from 12.6 to 8.9.

### Sialic acid contents of EBNs

**Fig. 2:**
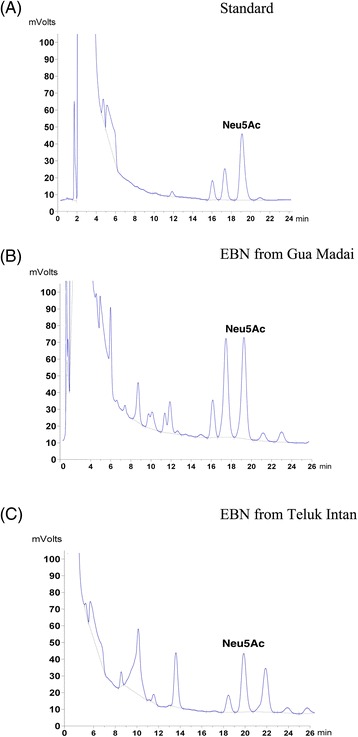
Fluorometric HPLC analysis of sialic acids in EBNs from Gua Madai and Teluk Intan. The chromatograms show the release of Neu5Ac with around 20 min retention time in **a** standard, **b** EBN from Gua Madai, and **c** EBN from Teluk Intan

HPLC analysis of the standard revealed that Neu5Ac would be released after around 20 min. Hence, following analysis of EBNs from Teluk Intan and Gua Madai showed that both EBN samples contain Neu5Ac with different concentrations. According to HPLC results, EBN from Gua Madai contained 6.7 mg/g of Neu5Ac, which was higher than 3.2 mg/gr in EBN from Teluk Intan. Figure 2 shows the chromatograms of fluorometric HPLC analysis of sialic acid in standard and both EBNs of this study.

### Modulation of the Rab5 protein

**Fig. 3:**
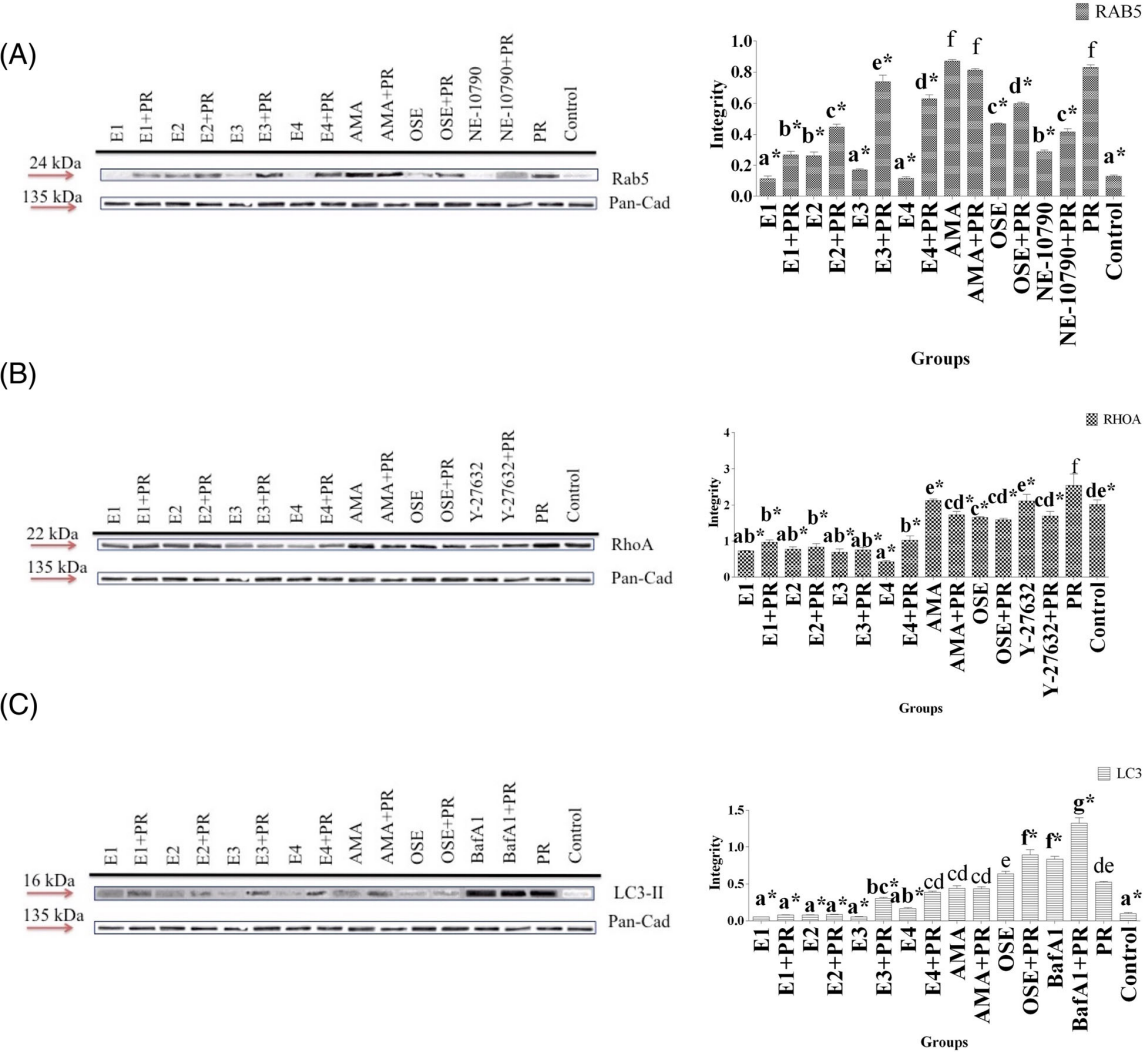
Effects of EBNs on Rab5 (**a**), RhoA (**b**), and LC3-II (**c**) proteins along with the acquired bands in the SDS-PAGE analysis. Alphabets show the Duncan analysis of the differences among the groups. * indicates the significant difference (*p* < 0.05) from the positive infected control (PR). E1: EBN from Teluk Intan with no enzymatic treatment, E2: EBN from Teluk Intan with Pancreatin F treatment, E3: EBN from Gua Madai with no enzymatic treatment, E4: EBN from Gua Madai with neuraminidase treatment, Ama: Amantadine hydrochloride, Ose: Oseltamivir carboxylate, PR: influenza A virus, A/Puerto Rico/8/1934 (H1N1)

Evaluation of Rab5 as a marker for early endosomes revealed that infection of MDCK cells with IAV caused significant (*p* < 0.05) increase in intracellular Rab5. All EBNs could significantly (*p* < 0.05) affect the endocytosis of the virus by decreasing of this protein (Fig. 3). However, the EBNs from Teluk Intan (EBN1 and EBN2) showed more efficiency compared to the ones from Gua Madai (EBN3 and EBN4). Regarding the enzymatic treatments, this study showed that the EBN with pancreatin F treatment (EBN2) had significant (*p* < 0.05) higher level of Rab5 in both infected and non-infected cells in comparison with EBN with no enzymatic treatment (EBN1). On the other hand, EBN with neuraminidase treatment (EBN4) showed significant (*p* < 0.05) lower level of Rab5 in only infected cells compared to EBN with no treatment (EBN3). In general, EBN extracts were more effective in reducing Rab5 than Rab5 inhibitor NE10790 (Fig. 3). Among the anti-influenza drugs, oseltamivir was also effective against the early endosomes trafficking and significantly (*p* < 0.05) decreased the amount of Rab5 in infected cells. This drug caused significant (*p* < 0.05) increase in Rab5 in the normal cells. In contrast to oseltamivir, amantadine did not affect Rab5 protein of neither infected nor normal cells (Fig. 3).

### Modulation of the RhoA protein

This study showed that IAV significantly (*p* < 0.05) increased the RhoA protein compared to the negative control. On the other hand, the results revealed that EBNs significantly (*p* < 0.05) decreased the RhoA in both presence and absence of the virus (Fig. 3). No significant difference was detected among different types of EBNs. Both EBNs from Gua Madai and Teluk Intan showed similar effects on RhoA protein in infected and non-infected cells. In addition, both enzymatic treated and untreated EBNs also reduced RhoA protein with no significant differences. Although treatment with the control drugs decreased RhoA, the treatments were not as effective as EBNs in reduction of this protein. The level of RhoA in amantadine and oseltamivir was significantly higher in both infected and non-infected cells in comparison with EBNs. These drugs could maintain the RhoA protein as the negative control level and only oseltamivir slightly reduced the RhoA level in normal cells. In addition, EBNs could significantly (*p* < 0.05) reduce RhoA protein more than Y-27632the control inhibitor of RhoA.

### Immunoblotting for LC3 localization

After analysis of LC3-II protein as the marker of autophagosomes, the results showed that EBNs significantly (*p* < 0.05) reduced the LC3-II protein in presence of the virus. EBNs from Teluk Intan showed significantly (*p* < 0.05) higher efficiency in reducing LC3-II protein level than the ones from Gua Madai (Fig. 3). However, pancreatin F treatment of EBN did not change this property of EBN in reducing of LC3-II protein. On the other hand, the EBN with neuraminidase treatment (EBN4) failed to reduce LC3-II protein in neither infected nor non-infected cells. In contrast to EBNs, oseltamivir significantly (*p* < 0.05) increased the amount of LC3-II protein in both infected and non-infected cells. Amantadine did not affect LC3-II protein in infected cells. However, this drug significantly (*p* < 0.05) increased the level of LC3-II protein in normal cells. BafA1 was used as the control inhibitor of the lysosome activity. Inhibition of the lysosomes with this pharmacological inhibitor significantly increased the amount of LC3-II protein and autophagosomes (Fig. 3).

### Actin cytoskeleton organization alterations

**Fig. 4:**
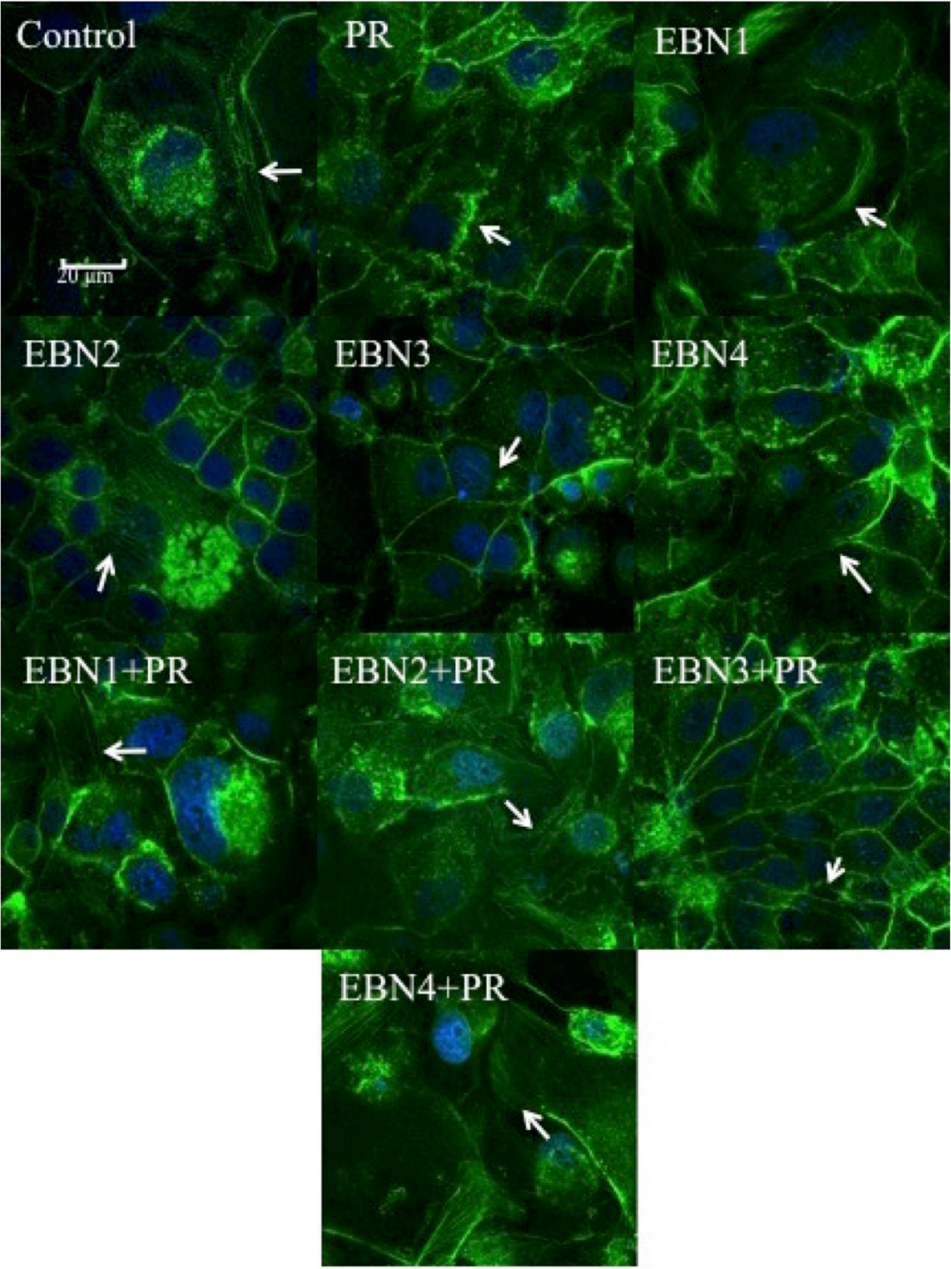
Reorientation of actin cytoskeleton in infected MDCK cells after treatment with EBNs detected by rhodamine 110 phalloidin staining. EBN1: EBN from Teluk Intan with no enzymatic treatment, EBN2: EBN from Teluk Intan with Pancreatin F treatment, EBN3: EBN from Gua Madai with no enzymatic treatment, EBN4: EBN from Gua Madai with neuraminidase treatment, Ama: Amantadine hydrochloride, Ose: Oseltamivir carboxylate, PR: influenza A virus, A/Puerto Rico/8/1934 (H1N1). The control staining shows the normal appearance of the actin filaments. The PR image shows the alteration of actin filaments in MDCK cells after infection with 100 TCID_50_/ml of the virus. The other images demonstrated the effects of EBNs in presence and absence of the virus. Bar: 20 μm. All images were taken at 100X oil immersion lens. The arrows show the actin filaments in each treatment

Immunofluorescent staining of the actin filaments in different treatments revealed that EBNs could reorient and normalize the actin filaments dissociation following virus infection. Apparently, IAV infection of the MDCK cells increased the actin stress fibers and impaired cytoskeleton filaments. As shown in Fig. 4, IAV increased the density of the actin filaments and change the shape of the cells. Treatment of infected cells with EBNs helped normalizing the cellular shapes and also reorienting the actin filaments (Fig. 4). In addition, the densities of actin filaments seem to be reduced by EBNs. In the absence of the virus, EBNs did not show any particular effect on the actin filaments.

### LysoTracker red staining analysis

**Fig. 5:**
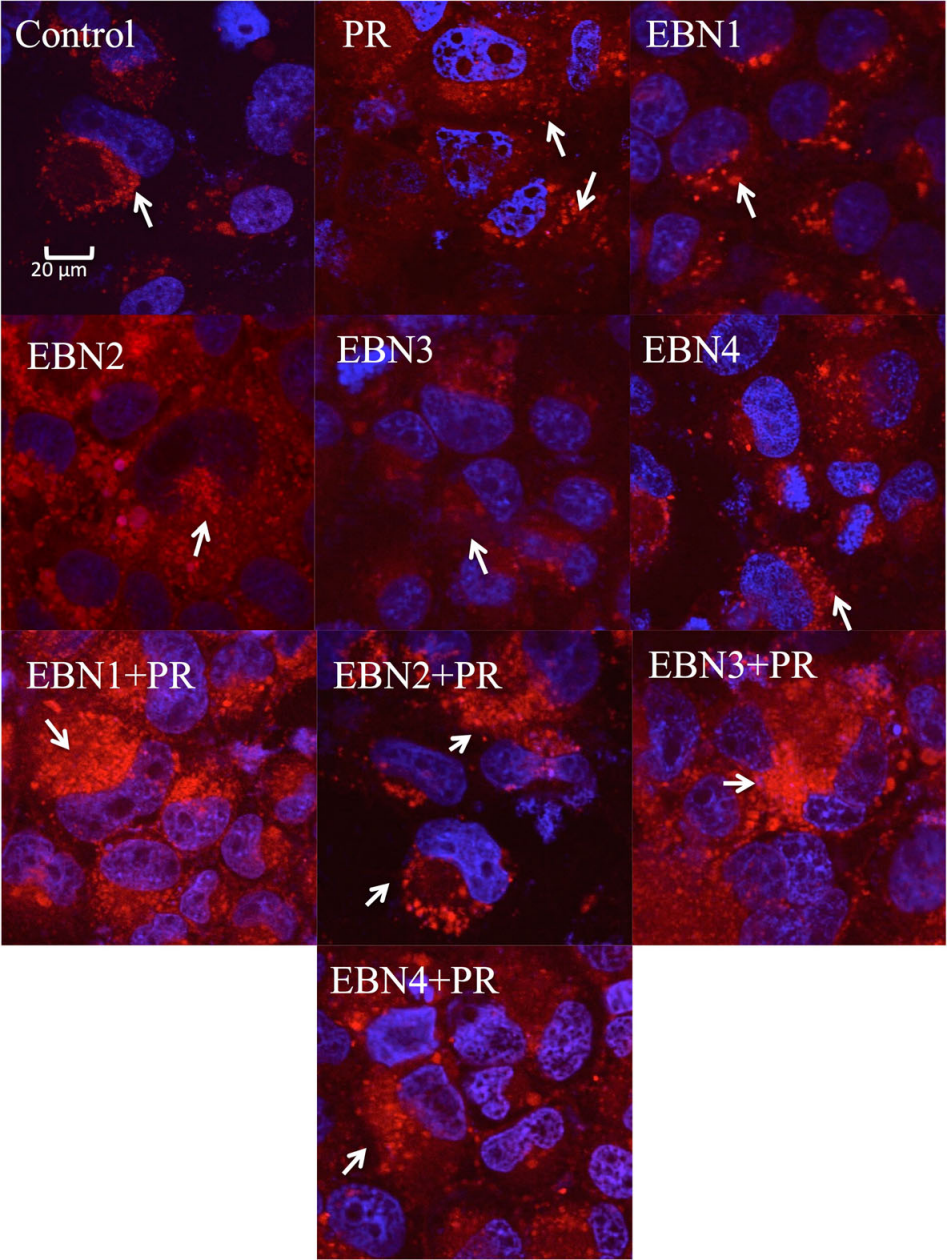
Increase of lysosomal activity in infected MDCK cells after treatment with EBNs detected by lysoTracker Red staining. EBN1: EBN from Teluk Intan with no enzymatic treatment, EBN2: EBN from Teluk Intan with Pancreatin F treatment, EBN3: EBN from Gua Madai with no enzymatic treatment, EBN4: EBN from Gua Madai with neuraminidase treatment, Ama: Amantadine hydrochloride, Ose: Oseltamivir carboxylate, PR: influenza A virus, A/Puerto Rico/8/1934 (H1N1). Images are the representatives of three replications for each treatment at 100 X oil immersion

Figure 5 shows the differences of intracellular lysosomal activityand the number of lysosomal bodies in normal and infected cells after treatments with different EBNs. IAV infection slightly increased the number of lysosomal bodies in comparison with the negative control. The results revealed that upon the treatment of both non-infected and infected cells with EBNs, the density and the number of lysosomes increased. While, most of the EBNs with different enzymatic treatments showed similar effects in increasing of lysosomal compartments, neuraminidase treatment of EBN seemed to slightly reduce this property of EBN in augmenting intracellular lysosomal activity (Fig. 5).

## Discussion

The results of our study showed that EBNs can act as effective as common antiviral agents against influenza virus albeit with distinct mechanism of action. In addition, some EBNs showed better efficacy and protective percentage against this virus than these drugs. While amantadine and oseltamivir had the highest efficacy when inoculated simultaneously with the virus, post inoculation of the EBNs after virus infection had the highest antiviral activity and percentage of protection against IAV. Hence, these results might indicate the mechanism of action of EBNs is not related to virus attachment to the host cell, but it might be relevant to its release process from the cell membrane. It is worth mentioning that this is the first study that investigates the antiviral activity of EBNs with different exposure methods.

In this study, EBNs originated from two different locations in Malaysia (Teluk Intan and Gua Madai) was treated with pancreatin F or neuraminidase during preparation. Regardless of enzymatic treatment, our results support the fact that depends on the origin, EBNs’ bioactivity can significantly change. Accordingly, the EBN from Gua Madai showed higher antiviral activity compared to the ones from Teluk Intan. Recently, Chua and his colleagues analyzed the metabolomics profiles of 152 EBNs with different origins and characteristics [12]. This study indicated that EBNs from different countries, coloration and production sites have significant variations in their metabolite compositions [12]. Previous studies also has shown that most of the proteins of the EBN is conjugated with different glycans such as O-glycans, glycosaminoglycans and N-glycans, particularly α2,3-*N*-acetylneuraminic acids (sialic acids) [24], which are able to inhibit the hemagglutination activity of different strains of influenza A viruses [8]. Hence, in this study we evaluated the Neu5Ac contents of our EBN samples. Apparently, the concentration of this sialic acid is correlated with antiviral activity of EBN against influenza, which caused higher potency of EBN from Gua Madai compared to the one from Teluk Intan. Hence, before introducing of this natural product as an antiviral agent, it is necessary to understand the mechanism of action and the metabolomics composition of the EBNs.

Two different enzymatic treatments were used in this study to elucidate the possible groups of active compounds in EBN against influenza virus. Pancreatin is a mixture of digestive enzymes that are secreted from pancreas with proteolytic, amylolytic and lipolytic activities. Previous studies showed that treatment of EBNs with this enzyme significantly increase the protein solubility, the degree of hydrolysis and bioactivepeptides concentration of the mixture [25]. In this study, the pancreatin F treatment of the Teluk Intan EBN significantly increased the antiviral activity of EBN. This result was not as predominant of previous study on the effects of pancreatin F treated EBN against influenza viruses [8], which might be the result of composition variation of EBNs used in these studies. Most likely, the EBN from Teluk Intan in our study has other active compounds in addition to amino acids or glycan residues, which are not affected by pancreatin F enzyme.

The other enzyme that was used in this study was neuraminidase. This is a glycoside hydrolysis enzyme that splits the glycosidic linkages of sialic acids [26]. As described before, sialic acids are one of the main components of EBN that correlate with antiviral activity of EBN. Hence, neuraminidase treatment of EBN can show the possibility of existence of other bioactive compounds in EBN against IAV. The results of our study also support the sialylglycoconjugates against IAV; however, even with neuraminidase treatment, EBNs still showed efficient antiviral activity in lower levels. Consequently, in addition to sialic acid residues, other bioactive compounds might inhibit influenza virus with direct or indirect mechanism of action. Recently, GC/MS and LC/MS metabolomic profiles of EBNs revealed that there are more than 78 metabolites belong to a wide range of chemical classes like organic acids, amino acids, sugar, sugar alcohol, fatty acid amides and steroids [12] with potential antiviral and antimicrobial activities. More studies are required to elucidate the effects of this compound against influenza virus.

During the influenza life cycle, this virus overcomes several barriers and manipulates the host cellular compartments to replicate and hide from the immune system. The endosomal system and the following autophagy process are two of the natural cellular pathways that are widely harnessed by influenza virus for its own benefits. After endocytosis or macropinocytosis of IAV inside the cell, the virus exploits these systems by inhibiting the endosomal maturation and changing in pH to release the vRNP into the cytoplasm for replication. While the virus inhibits the lysosomal degradation of the late endosomes and formation of the autolysosomes, it induces the formation of the autophagosomes to accelerate the replication and manipulate the host immune responses [6]. The virus also can trigger the autophagic cell death or regulate the apoptosis by maneuvering the autophagic system [27, 28]. Hence, inhibiting or interfering with these mechanisms of the virus can be a promising strategy for antiviral development. The results of this study revealed that EBNs could affect the early endosomes and trafficking of these vesicles through manipulation of actin filaments. This can be demonstrated by the decrement of RhoA as the regulator of the actin polymerization, and remodeling the actin filaments after the treatment (Figs. 3 and 4). In addition, it has been showed that decreased of Rab5 could efficiently reduce the influenza infection [29]. Actin filaments also showed an important role in trafficking of the initial viral particles in polarized epithelial cells [30]. Previous studies have shown that inhibition of actin filament polymerization by cytochalasin D treatment decreased the viral entry to the apical phase of polarized epithelial cells [31]. However, this inhibitor was not effective in non-polarized cells or entering the virus from the basolateral phase of the polarized epithelial cells. Hence, although the amount of viral entry can be interrupted by manipulation of the actin filaments, the viral infectivity may not be affected by this mechanism [30, 31].

Despite the effects of EBNs on Rab5, RhoA and actin filaments, this study showed that pre treatment and co treatment of influenza virus with EBNs is not effective in reducing viral load. This result suggests that EBNs do not have efficient activity in inhibiting the initial stages of viral entry probably due to the high concentration of epidermal growth factor (EGF) in EBNs [17]. Previous study has shown that the attachment and internalization of the IAV can be promoted by the activation of EGF receptors [32]. Hence, in contrast to bioactivity of EBN in decreasing the Rab5 and alteration of actin cytoskeleton, a metabolite-like EGF can facilitate the entrance of the virus. This effect may have been emphasized by enzymatic treatment of EBN since pancreatin F treatment of the EBN (EBN2) lead to augmentation of Rab5 compared to the negative control. Hence, additional studies are required to determine the bioactive compounds of the EBN against Rab5 protein and study the effects of EBN on initial stages of influenza life cycle with control of the content EGF.

Regarding the autophagy mechanism, EBN could efficiently decrease the LC3-II protein and increase the lysosomal degradation. LC3 is a protein that distributed throughout the cytoplasm. However, after cleavage and conjugation with phosphatidyl ethanolamine, it will be localized on the surface of autophagosomes until the fusion with lysosomes [33]. Hence, LC3-II level can be a good marker for monitoring of autophagosomes accumulation [34]. Previous study has shown that inhibition of lysosomal proteases increased the LC3-II level during influenza infection [35]. Thus, it can be concluded that the effect of EBN is through increase of lysosomal degradation and decrease of autophagosomes accumulation. In this way, the intracellular accumulated viruses can be degraded efficiently. During the influenza life cycle, autophagy serves as an anabolic pool for virus assembly and this virus triggers accumulation of autophagosomes through M2, HA, and NS1 proteins [36]. Previous study has shown that the inhibition of autophagy would decrease the influenza virus titer [37]. However, this kind of inhibition does not affect the replication of a highly pathogenic strain like H5N1 and only slightly ameliorate the disease [38]. Further studies are required to evaluate the efficacy of EBN against H5N1 and the autophagy mechanisms against this virus.

## Conclusions

Traditional medications and different natural products have been of interest to be used for antiviral treatments. Hence, these days a considerable amount of study is underway on the potential benefit of EBN as therapeutic agent. Although, some EBN extracts showed SI less than 10, other different ingredients in the EBN extracts my give different ways of antiviral activity. Thus, we cannot ignore the other EBNs simply just because of low SI. The results procured in this study support the potential of EBNs as supplementary medication or alternative to antiviral agents to inhibit influenza infections. Evidently, EBNs can be a promising antiviral agent; however, these natural compounds should be screened for their metabolites. For the first time, this study showed that EBNs can inhibit the autophagy process during influenza virus life cycle, which can efficiently result in decrease in viral replication.

## Abbreviations

ANOVA: Analysis of variance
ATCC: American Type Culture Collection
CC_50_: 50% Cytotoxic Concentration
CPE: Cytopathic effect
CRBC: Chicken red blood cells
DAPI: 4′, 6-diamidino-2-phenylindole
DMEM: Dulbecco’s Modified Eagle’s Medium
EBN: Edible bird nest
EGF: Epidermal growth factor
FBS: Fetal bovine serum
GCMS: Gas chromatography mass spectrometric
HA: Hemagglutination assay
HPLC: High performance liquid chromatography
IAV: Influenza A virus
IC_50_: 50% Inhibitory concentration
LCMS: Liquid chromatography mass spectrometric
MDCK: Madin-Darby Canine Kidney
MNCC: Maximum non cytotoxic concentration
Neu5Ac: 5-*N*-acetylneuraminic acid
NO: Nitric oxide
TCM: Traditional Chinese medicine
TPCK: Tosylamide phenylethyl chloromethyl keton-treated trypsin
